# Corneal structure and transparency

**DOI:** 10.1016/j.preteyeres.2015.07.001

**Published:** 2015-11

**Authors:** Keith M. Meek, Carlo Knupp

**Affiliations:** Structural Biophysics Research Group, School of Optometry and Vision Sciences, Cardiff University, Maindy Road, Cardiff CF24 4HQ, UK

**Keywords:** Cornea, Transparency, Structure, Collagen, X-ray, Three-dimensional electron microscopy, Theoretical modelling

## Abstract

The corneal stroma plays several pivotal roles within the eye. Optically, it is the main refracting lens and thus has to combine almost perfect transmission of visible light with precise shape, in order to focus incoming light. Furthermore, mechanically it has to be extremely tough to protect the inner contents of the eye. These functions are governed by its structure at all hierarchical levels. The basic principles of corneal structure and transparency have been known for some time, but in recent years X-ray scattering and other methods have revealed that the details of this structure are far more complex than previously thought and that the intricacy of the arrangement of the collagenous lamellae provides the shape and the mechanical properties of the tissue. At the molecular level, modern technologies and theoretical modelling have started to explain exactly how the collagen fibrils are arranged within the stromal lamellae and how proteoglycans maintain this ultrastructure. In this review we describe the current state of knowledge about the three-dimensional stromal architecture at the microscopic level, and about the control mechanisms at the nanoscopic level that lead to optical transparency.

## Highlights

• We are coming close to understanding how its complex structure governs the shape and biomechanical behaviour of the cornea.
• Transparency arises from the arrangement of collagen fibrils and the presence of crystallins within the stromal cells.
• Fibril positions at any instant are maintained by the arrangement of proteoglycans around and between them.
• Transparency loss in pathology or following surgery can arise from a combination of many different structural alterations.

## 1 Introduction

The transparent cornea forms the anterior portion of the outer casing of the eye and has the dual functions of protecting the inner contents of the eye as well as providing about two thirds of the eye's refractive power. The human cornea is composed of five layers, an overlying epithelium beneath which is a fibrous meshwork called Bowman's layer. The bulk of the tissue is constituted by the stroma, a collagen-rich central layer that comprises nearly 90% of the thickness of the cornea, and beneath this lies Descemet's membrane which supports the single layer of endothelial cells lining the posterior cornea. Other species have been reported to have certain differences in this construction, particularly with respect Bowman's layer and Descemet's membrane (Hayashi et al., 2002) but nevertheless, all these corneal layers need to be transparent. In normal corneas most of these are so thin that light scattering is minimal. For example, in humans, Bowman's layer and Descemet's membrane, both collagenous tissues like the stroma, together contribute less than 4% to the total corneal thickness. The corneal epithelium, on the other hand, is about 53 μm deep (Reinstein et al., 2008) and thus constitutes about 10% of the corneal thickness. Its transparency is a result of the homogeneity of the refractive index of all its constituent cells (Dohlman, 1971). In this review, we will concentrate on the structure and transparency of the corneal stroma. However, it should be noted that in a number of corneal pathologies, changes in one or more of the other layers can lead to increased light scattering and consequent loss of corneal transparency.

In most mammals, the cornea is the only tissue requiring considerable tensile strength coupled with a perfectly defined shape and optical clarity. For nearly a century it has been realised that these properties may derive from the arrangement of the constituents of the stroma (Eisler, 1930, Kolmer and Lauber, 1936). About 50 years ago the basic ultrastructure had been described (Jakus, 1962) and the principles behind corneal transparency finally elucidated (Maurice, 1957, Hart and Farrell, 1969, Benedek, 1971) so corneal research focussed more on other areas. However, with the advent of a host of new clinical techniques ranging from laser refractive surgery to corneal cross-linking, interest in how the shape, strength and transparency of the cornea is achieved and maintained has grown. The past decades have seen the emergence of a number of exciting new methodologies that have allowed us to gain considerable insight into how this structure forms, how it is maintained, and how it achieves the biomechanical and optical properties required for a functional cornea. These new discoveries are supporting efforts by surgeons and others to improve clinical techniques, and by bioengineers and computer modellers to understand and predict the behaviour of the tissue after surgical interventions. They also underpin efforts to develop artificial biological corneal replacements. In this review, we describe our current understanding of the structure of the corneal stroma at all hierarchical levels. We review the latest advances demonstrating how the microscopic structure controls the shape of the cornea, how the nanoscopic arrangement of collagen fibrils ensures corneal transparency, and how this unique fibrillar arrangement arises and is maintained.

## 2 Stromal micro- and nano-structure

The corneal stroma has three primary non-aqueous constituents: collagens, proteoglycans and cells. It also contains specialised glycoproteins (Labat-Robert and Robert, 2012, Wall et al., 1988, Cooper et al., 2006) and, of course, ions that play an important role in organising the collagen fibrils in order to maintain transparency (Kostyuk et al., 2002, Regini et al., 2004). Many of the characteristics of corneal collagen and its structural organisation have been described elsewhere (Meek and Quantock, 2001, Meek and Boote, 2009, Meek, 2009) so here we give only a brief overview and a more extensive update of the most recent findings.

### 2.1 Lamellae

It has long been known that, at the microscopic level, the collagen in the stroma is laid down within lamellae. These structures are of variable thickness, in humans typically up to 0.2 mm broad and 2 μm thick (Polack, 1961, Komai and Ushiki, 1991). At the centre of the human cornea there are approximately 200 lamellae through the thickness, and the packing density is higher in the anterior lamella than in the posterior ones (Bergmanson et al., 2005). These anterior lamellae are highly interwoven (Radner et al., 1998) and most appear to insert into Bowman's layer (Morishige et al., 2006). The mid-stromal lamellae are also highly interlaced (Radner and Mallinger, 2002). The posterior lamellae in the central cornea are more hydrated and are believed to have less interlacing, lying on top of each other like the layers in plywood. The posterior stroma can swell easily whereas the more interwoven anterior cannot (Müller et al., 2001).

With the advent of new corneal surgical techniques aimed at avoiding penetrating keratoplasty (such as Deep Anterior Lamellar Keratoplasty, Descemet's Stripping Endothelial Keratoplasty and related procedures) it is becoming common to inject air into the cornea to separate the endothelium and Descemet's membrane (pneumodissection) in order to make the surgery easier to perform. For some time, ophthalmologists have been aware that when a so-called “big bubble” is induced, part of the posterior stroma often adheres to the Descemet's membrane, and that there is, therefore, a natural cleavage plane in the stroma about 10 μm above Descemet's membrane (Jafarinasab et al., 2010, McKee et al., 2011). A study of the different types of big bubble that can be formed led Dua et al. (2013) to propose, somewhat controversially, that the most posterior region of the stroma below the final layer of keratocytes is distinct, and should therefore be classed as a separate layer, which they termed Dua's layer or pre-Descemet's layer (PDL). This region was shown to be intimately related to the trabecular meshwork and, like that structure, to be rich in type VI collagen (Dua et al., 2014). Barring an anchoring zone of interwoven collagen fibrils at the Descemet-stroma interface, Schlötzer-Schrehardt et al. (2015) found no evidence for the existence of a distinctive acellular PDL in the human cornea. They concluded that the intrastromal cleavage plane after pneumodissection was a result of the specific arrangement of keratocytes across the cornea in this region, and that it is determined by the intra-individually and inter-individually variable distances of keratocytes from Descemet's membrane. Although our own observations of this region, using X-ray scattering, have revealed no differences in the collagen fibrillar arrangement, there is also an elastic fibre network within the stroma (McIlroy, 1906, Alexander and Garner, 1983). We have shown in the human cornea that these elastic fibres, though present throughout most of the stromal depth, are concentrated below the posterior-most keratocyte layer (unpublished results). Notwithstanding these findings, at the time of writing the jury is still out, although it cannot be denied that the way injected air moves through the stromal lamellae in this region has very important implications for big bubble surgical techniques.

**Fig. 1:**
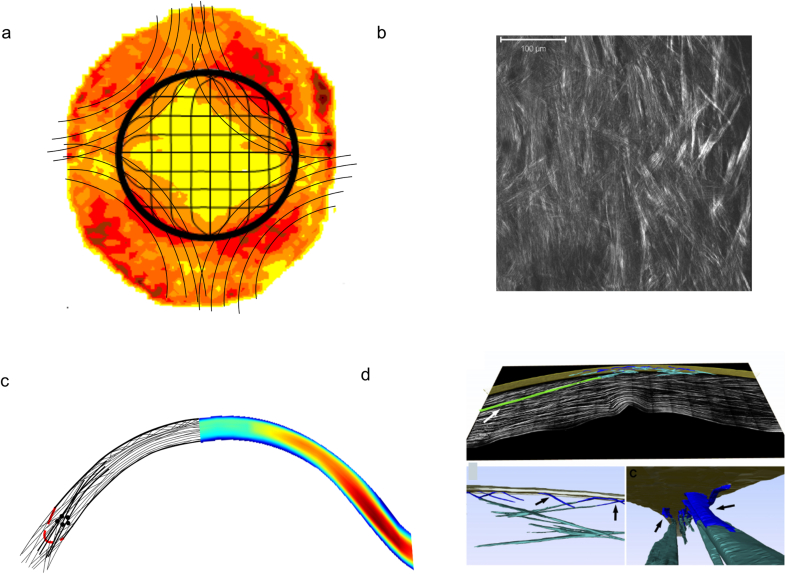
Simplified model of the principal lamellar orientations in the human cornea. (a) and (c) are based on X-ray data; (b) and (d) are based on second harmonic generated (SHG) microscopy. (a) and (b) are en face views from the front of the cornea. The X-ray data (a) show the model (black lines) superimposed on the distribution of preferentially aligned collagen lamellae and indicates preferred lamellar orientations in the inferior-superior and nasal-temporal directions, predominantly in the posterior stroma. Many of these seem to become part of a circum-corneal annulus at the limbal region. In addition, anchoring lamellae with larger collagen fibrils are thought to enter the stroma in alignment with the extraocular musculature. The lines in the model show the predominant orientation of lamellae, not the actual course of individual lamellae. SHG microscopy from the posterior stroma (b) reveals an interwoven lattice of lamellae, such that adjacent lamellae are not orthogonal; thus no preferred orientation of collagen is obvious in the central cornea using this technique. (c) and (d) are views through the thickness of the stroma. In (c) the X-ray data are shown on the right. The corresponding model on the left suggests that lamellae are more interwoven anteriorly, particularly in the central 4 mm zone. Outside this zone, interweaving is more prominent in deeper stromal layers also. At the limbus (indicated by broken red line), the circumcorneal annulus appears in cross-section (represented by black circles) and anchoring lamellae appear to enter the stroma from the deeper layers and gradually move towards the surface. SHG microscopy (d) also demonstrates the highly interwoven anterior lamellae, which are often referred to as fibres using this imaging modality. A 3-D reconstruction shows bow spring fibres (blue), anchoring fibres inserting from the limbus (green), and the highly intertwined anterior fibre meshwork (teal) near Bowman's layer (gold). (d) is reproduced from Winkler et al. (2011) with permission of the copyright holder.

Moving from the central cornea towards the limbus the human cornea thickens; hydration is fairly constant in this direction in the pig and the cow (S. Hayes, unpublished results; Ho et al., 2014) and, although water distribution from central cornea to limbus is not known for human corneas, it seems likely that alterations in tissue thickness are due to an increase in the amount of collagen in the peripheral stroma (Aghamohammadzadeh et al., 2004, Boote et al., 2011, Henriksson et al., 2012). In addition, stromal interweaving in the peripheral cornea seems to extend to the deeper posterior lamellae (Radner et al., 1998, Abass et al., 2015). The posterior limbus accommodates a circum-corneal annulus (Fig. 1a) in which elastic fibres run parallel to collagen fibres (Kamma-Lorger et al., 2010). The exact arrangement of this elastic system in the rest of the cornea remains to be elucidated, but current work in our group has revealed that many fibres seem to originate in the limbus, forming sheets at the edge of the cornea which then split up and extend as microfibrils, possibly oxytalan or elaunin (Alexander and Garner, 1983), across the stroma (White et al., 2014). This elastic system may have an important biomechanical role but it is unlikely to influence transparency in the centre of the normal cornea.

Although preferred lamellar orientations in the human corneal stroma were originally noted by Weiner (1912) and Kokott (1938), during the last decade or so, X-ray scattering pioneered by our group has quantified this arrangement (for review see Meek and Boote, 2009). Across the anterior stroma, lamellae are highly interwoven (Radner et al., 1998) and randomly directed when observed *en face* (Abahussin et al., 2009). Collagen lamellae in the deeper stroma (Fig. 1a) have preferred directions, which appear to be close to the inferior-superior and nasal-temporal directions (Meek et al., 1987, Aghamohammadzadeh et al., 2004, Abahussin et al., 2009, Kamma-Lorger et al., 2010). X-ray scattering has also suggested the presence of a network of collagen lamellae that enter the cornea close to the inferior, superior, nasal and temporal positions, and which probably originate in the adjacent sclera (Aghamohammadzadeh et al., 2004). This network shows mirror symmetry between left and right eyes (Boote et al., 2006) and seems to contain collagen fibrils with a larger diameter to those in the central cornea (Boote et al., 2011). The arrangement of these putative structures seem to correspond with the sheer stress distribution in the peripheral cornea (Antony, 2015). We have named them “anchoring lamellae” as they may play a role in maintaining the structural integrity of the cornea and sclera (Fig. 1a).

The same techniques have been used to quantify the collagen mass density and lamella inclination angles with respect to the corneal surface as a function of depth and position across the cornea and limbus. This confirmed earlier data (Boote et al., 2011) demonstrating the presence of larger diameter fibrils in the peripheral stroma and led to the model of the nasal-temporal cross-section shown in Fig. 1c. It seems that the anchoring lamellae enter the cornea from the sclera, and may work their way towards the corneal surface without reaching the central optical zone (Abass et al., 2015, Winkler et al., 2011).

The X-ray technique does not allow direct imaging of the lamellar structures; the arrangement of lamellae has to be inferred from the diffraction patterns. Furthermore, the X-ray data represent bulk averages throughout the tissue being sampled so, for instance, it is not possible to obtain information pertaining to adjacent lamellae using this approach. Over the last decade there has been an upsurge in the use of non-linear microscopy, in particular second harmonic generation imaging, to provide qualitative (Han et al., 2005, Morishige et al., 2006, Teng et al., 2006, Bueno et al., 2011) and quantitative (Mega et al., 2012, Tuer et al., 2012, Ghazaryan et al., 2013) information about the lamellar organisation within the cornea, a technique that, unlike X-ray scattering, has the potential to be used in vivo (Latour et al., 2012). It has also been claimed that second harmonic generation signals can be used to measure collagen fibril diameters down to 30 nm (Bancelin et al., 2014). The technique was recently used to show that mammalian corneas do not have an orthogonal arrangement of adjacent lamellae, whereas non-mammalian vertebrate corneas share a common orthogonal collagen structural organisation, which suggests that they have a divergent evolutionary background (Winkler et al., 2015).

Many of the above studies have involved animal corneas. In human corneas, non-linear microscopy has been used to quantify the lamella arrangement at different corneal depths, where the authors could confirm that in the posterior stoma, lamellae occur preferentially in orthogonal directions (Lombardo et al., 2014). Also in human corneas, Jester et al. (2010) were able to reconstruct the lamellar organisation through the full thickness of the cornea from limbus-to-limbus. The reconstructions demonstrated the interweaving of the anterior lamellae and their bow spring-like insertions in and out of Bowman's layer (Fig. 1d). Quantitative analysis later revealed that the insertion angles were, on average, about 19° (Morishige et al., 2011) and that this bow spring-like arrangement provides structural support to maintain the shape of the anterior corneal surface (Winkler et al., 2013). These quantitative data from the depth-dependent out-of-plane organisation of lamellae have helped to explain the experimentally measured depth dependence of the mechanical properties of the human cornea (Petsche and Pinsky, 2013).

Both scanning electron microscopy (Radner et al., 1998) and non-linear SHG microscopy (Winkler et al., 2015) have shown that, in the human cornea, there is no orthogonal arrangement between adjacent lamellae (Fig. 1b), not even in the posterior stroma. It should be pointed out that this does not contradict the X-ray results, which simply indicate that, over many posterior lamellae, there are preferred mutually orthogonal lamellar directions, but say nothing about angles between adjacent lamellae.

The lamellae have long been known to consist of narrow, parallel collagen fibrils embedded in a proteoglycan-rich hydrated matrix. What has often been overlooked is that, even within the in vivo cornea, on a micron scale these fibrils are probably not straight but rather have a crimp as in other connective tissues (Grytz and Meschke, 2009). There is a distribution of crimp angles, which can be altered under mechanical load. This distribution may be related to the anterior-posterior angle in which a lamella crosses the cornea, but this has yet to be determined. However, it has been postulated that this crimping plays an important role in the biomechanical response of the cornea (Liu et al., 2014). In fact, collagen crimp, in combination with elastic fibres in the peripheral cornea, may explain why this region is more pliable and absorbs intraocular pressure fluctuations (Boyce et al., 2008), thus preventing deformation of the optically important central cornea.

The lamella arrangement within the cornea helps to maintain the overall shape of the tissue and is also responsible for some optical properties such as corneal birefringence. However, it plays no significant role in corneal transparency – this property is defined at the microscopic level by the absence of scatter by corneal cells or by other structures that could cause local changes to the refractive index, and at the nanoscopic level, by the absence of pigments or blood vessels (in most species), and by the structure and organisation of the collagen fibrils within the lamellae (Meek et al., 2003).

### 2.2 Collagen fibrils

**Fig. 2:**
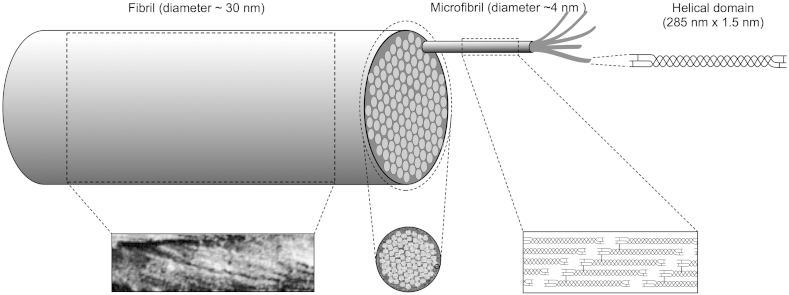
Structural hierarchy in corneal collagen (not to scale). Three helical alpha chains are supercoiled to produce the collagen triple helix molecule (top right). These molecules self-assemble in a staggered axial array (bottom right) to form microfibrils consisting of five molecules which in turn coil together to form the 30 nm diameter collagen fibrils seen in the electron microscope. The micrograph bottom left is reproduced from Ottani et al. (2002), with permission of the copyright holder, and shows the coiled microfibrils within the collagen fibril; the micrograph bottom middle is reproduced from Baldock et al. (2002), with permission of the copyright holder, and shows the microfibrils in cross-section within the collagen fibril.

Collagen fibrils within the cornea are narrower than in many other connective tissues and this is an important factor for transparency, which is a function of the diameter (Hart and Farrell, 1969). There are about 300–400 triple-helical molecules within the cross-section of a fibril, depending on the species (Meek and Leonard, 1993, Holmes and Kadler, 2005), which are arranged axially with the typical 67 nm D-periodic stagger (Meek and Holmes, 1983) with gaps between the N- and C-termini of consecutive molecules (Fig. 2). Of these molecules, about 30–40 in the bovine cornea are type V collagen, the rest are type I. The incorporation of type V into hybrid fibrils plays an important role in limiting the number of molecules that can self-assemble within the fibril (Birk et al., 1986).

Corneal collagen fibrils, as the principal load-bearing constituents of the lamellae, have to resist the tensile forces due to the intraocular pressure and protect the inner ocular tissues from external trauma while at the same time remaining narrow to allow transparency of the tissue; these are competing requirements. This appears to be achieved by a number of structural complexities, most of which have been studied in bovine corneas. The collagen molecules have left-handed helical polypeptide chains in a right-handed superhelix. At the supra-molecular level, the initial building block is a 4–8 nm wide microfibril (Baldock et al., 2002) with a left-handed twist of the constituent molecules (Fig. 2). Microfibrils have a right-handed twist within the fibrils (Orgel et al., 2006), and are stabilised by covalent intermolecular and intramolecular crosslinks (Yamauchi et al., 1996). There are about 70 microfibrils within each fibril (Holmes and Kadler, 2005) which are tilted by about 15° to the fibril long axis (Holmes et al., 2001) and this reduces the usual 67 nm axial periodicity seen in tendon and sclera, to closer to 65 nm (Meek et al., 1981, Marchini et al., 1986, Yamamoto et al., 2000). Many other microscopical techniques also reveal the presence of this microfibrillar substructure within corneal collagen fibrils (Meek, 2009). It has been suggested that in such super-helical structures, it is likely that there is a central core of a different nature (Raspanti et al., 2011), though this has not yet been demonstrated in the cornea. This cross-linked rope-like structure with alternating twist at each hierarchical level conveys considerable strength to the narrow corneal fibrils, and is in contrast to the fibrils in the adjacent sclera, which are correspondingly wider and therefore have a more parallel arrangement of microfibrils within them (Yamamoto et al., 2000).

The surfaces of corneal collagen fibrils have been studied by both scanning electron microscopy and atomic force microscopy. It appears that fibrils have surface dips or grooves in the gap zone compared to the overlap zone, explained by the 4:5 ratio of molecular density in the two zones. In human corneas, the depth of the grooves in the gaps is about 0.23 nm (Meller et al., 1997). These gaps play significant roles in different connective tissues. In bone, they are the nucleation sites for the calcification process. In cornea, they may help to accommodate the non-collagenous extensions of the component type V molecules and thus play a role in limiting fibrillar growth by molecular accretion (see later). They are also a primary binding site for dermatan sulphate-containing proteoglycans (Scott and Haigh, 1988), which, as we will also see later, are necessary for preventing adjacent fibrils from fusing together (Chen and Birk, 2013). When proteoglycans have their attached glycosaminoglycans removed enzymatically, the surface gap depth increases to about 1.5 nm (Yamamoto et al., 2002).

### 2.3 Proteoglycans

Early researchers were aware that the collagen fibrils in the corneal stroma were embedded in some kind of matrix gel. Virchow (1905) was the first to realise that this interfibrillar gel was of importance to corneal transparency and Meyer (1948) reported that the gel, by then known to contain mucopolysaccharides, maintained transparency by regulating its water content, and discovered that it contains dermatan sulphate, keratan sulphate and chondroitin sulphate (Meyer et al., 1953). The term mucopolysaccharide was therefore replaced by the term glycosamonoglycan (GAG) to better describe the chemical composition. During the 1950s the glycosaminoglycans were discovered to be attached to protein cores, hence the term proteoglycan was coined to describe the whole molecule, GAG and protein (for review see Yanagishita, 1993). In the late 1980s proteoglycans were given specific names depending on their GAG chains and on the nature of their protein cores. The normal adult human stroma contains four small leucine-rich proteoglycans: decorin, lumican, keratocan and mimecan. The first contains dermatan sulphate and chondroitin sulphate, while the final three contain keratan sulphate GAGs. Two other proteoglycans associated with the cornea are biglycan (containing dermatan sulphate) and fibromodulin (containing keratan sulphate). Biglycan occurs in the epithelium and in the stroma of some pathological corneas (Funderburgh et al., 1998); fibromodulin plays a major role in development of the peripheral cornea but its presence in the normal cornea is uncertain (Chen et al., 2010). The protein cores of these proteoglycans contain leucine-rich repeats which, in the central part of the molecule, form a curved solenoid structure with convex and concave faces flanked by cysteine-rich domains on two sides. C-terminal capping motifs in decorin, lumican and keratocan have an “ear repeat”, a leucine-rich repeat that extends outward from the convex face and probably helps to maintain protein conformation and collagen-binding ability (for review see Chen and Birk, 2013). The individual proteoglycan core proteins are thought to bind to collagen fibrils at specific axial sites along the collagen fibrils (Meek et al., 1986, Scott and Haigh, 1988).

## 3 Measurement of corneal transparency

In many publications, corneal transparency ex-vivo has been qualitatively assessed by laying the cornea on top of some text or on top of a grid, and making a subjective assessment of how easy it is to read or to see the grid through the tissue. This technique may be useful for comparative purposes as it is simple and quick, and we have used it ourselves (Mi et al., 2011). However, Maurice (2001) pointed out that this can be an extremely insensitive test, since very different results can be observed by either laying the cornea on the grid or holding the cornea in front of the eye and then observing the grid. He concluded that the test material should not be in contact with the grid or text but should be held in front of the eye.

Quantitative measurement of corneal light transmission requires passing a defined beam of light through the tissue and detecting how much is transmitted without absorption or scattering. It is important to avoid the effects of refraction and reflection and this can be achieved by surrounding the tissue in a medium with a similar refractive index to that of the cornea. It is also important to ensure that the cornea is maintained under tension, as it is usually necessary to remove the cornea from the globe to make the transmission measurement. Otherwise, when the tension due to the intraocular pressure is released, it seems that the resulting undulations of the lamellae lead to increased light scatter (McCally and Farrell, 1977, Kostyuk et al., 2002). This may be less of a problem in the human cornea when excised with a scleral ring, as these corneas tend to retain their shape much better than many other species, though specific testing on excised human corneas has not been carried out. Finally, one must ensure that the detector has a small acceptance angle so as not to record any forward scattered light (Farrell et al., 1973).

**Fig. 3:**
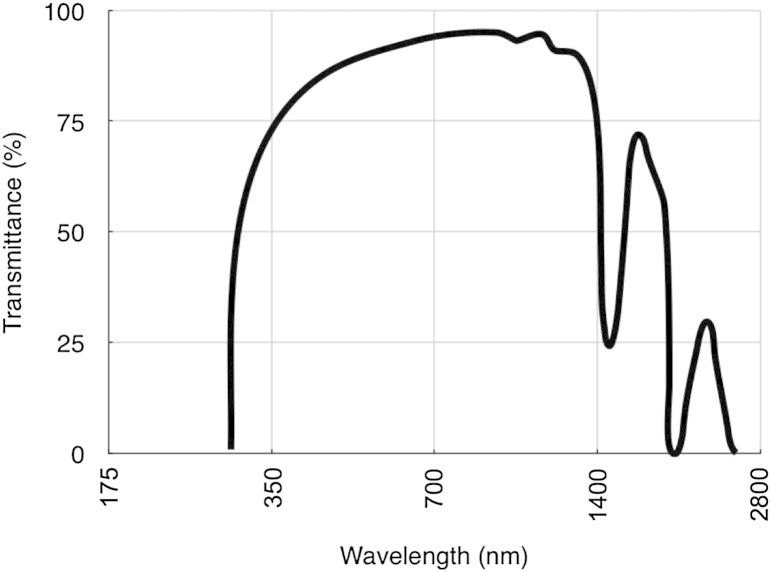
Transmittance through the human cornea as a function of wavelength. Reproduced in part from Boettner and Wolter (1962) with permission of the copyright holder.

The two most commonly used techniques to measure corneal transparency *ex vivo* are tungsten light-based or laser-based bench-top optical systems (Farrell et al., 1973, Christens-Barry et al., 1996, Parekh et al., 2014), or spectrophotometry (Cejka et al., 2010, Doutch et al., 2008). To our knowledge, one of the first attempt to measure the transparency of isolated human corneas was carried out by Boettner and Wolter (1962), using spectrophotometry. They examined transmission from 220 nm in the ultraviolet (UV) region, through to 2800 nm in the infrared region. There was very little transmission of UVB and UVC through the cornea because the epithelium and stroma contain specialist proteins and vitamins that are thought to absorb much of this radiation and thus protect the cornea and the inner contents of the eye. Most UV absorbance occurs in the anterior corneal layers (Kolozsvári et al., 2002). Boettner and Wolter (1962) found that the corneal transmittance rose rapidly from 300 nm, reaching 80% at 380 nm and more than 90% between 500 nm and 1300 nm. Two drops in infrared transmitted intensity were noted at 1430 nm and 1950 nm which are caused by water absorption (Fig. 3). Lerman (1984) and Olsen (1982), using slit lamp source and a fibre optic coupling from the microscope to a detector, found a decrease in light transmission through the human cornea with age whereas Van Best et al. (1988), using a xenon light source and a photodiode detector, measured corneal transmission in whole human eyes and found that transmission in the centre of the visible region was independent of age. This was extended into the near infrared (Beems and Van Best, 1990) where, once again, no age differences were noted, but, unlike Boettner and Wolter (1962), between 600 nm and 1000 nm, transmission values above 95% were recorded. Until recently, only our group has measured light transmission away from the optical axis. Using spectrophotometry we showed that, within the visible spectrum, transmittance decreases outside the central 4 mm pre-pupillary cornea (Doutch et al., 2007). Theoretical calculations of light transmission (Doutch et al., 2008) attributed this to the increase in fibril radius that occurs in the periphery of the cornea (Boote et al., 2003). Interestingly, in both cows and humans, the highest transmission in the pre-pupillary cornea is slightly displaced from the optical centre, but the functional reason for this is not known (Doutch et al., 2007). Although Boettner and Wolter (1962) showed that little UV penetrates the cornea, some does reach the deeper stroma (Zigman, 1993), potentially damaging the sensitive endothelial cells. We found that UV transmission through the cornea decreases from the centre to the periphery, and attributed this to a combination of scattering and absorption (Doutch et al., 2012). In this context it is noteworthy that the endothelial cells in the central 3 mm of the cornea suffer increased age-related (probably ultraviolet-induced) oxidative damage compared to cells further out (Mimura and Joyce, 2006, Joyce et al., 2009). With the introduction of UVA/riboflavin corneal crosslinking, the penetration of UVA in the cornea has been re-examined (Lombardo et al., 2015). The authors found that UVA transmission decreases almost linearly from the centre to the periphery in all meridians, and this could have implications for the effects of crosslinking treatments.

One of the early attempts to measure light scatter in vivo was by Rispoli et al. (1978) which was refined by Barbaro et al. (1988). This was based on the use of an optical fibre and on the assumption that the eye can be considered as a “black body” in physiological conditions under certain lighting conditions. Since then, techniques based on measuring the scattered rather than the transmitted light have been developed. These include Scheimpflug photography (Smith et al., 1990, Beckman Rehnman et al., 2011, Rozema et al., 2011), video pachometry using a slit lamp (Lohmann et al., 1992, Connon et al., 2003, Patel et al., 2007), confocal microscopy (Jester et al., 2001) and optical coherence tomography (Wang et al., 2004, Hoffart, 2013). Most of these techniques detect back-scattered light. Forward scatter has been measured using confocal microscopy (Ginis et al., 2009), by making direct measurement in excised corneas (De Brouwere et al., 2008) or by using stray light techniques on humans in vivo (Van den Berg et al., 2013, Patel et al., 2008, Rozema et al., 2011) for which commercially equipment is available. In so-called Direct Compensation Stray Light measurements, a test field consisting of a dark circle split into left and right halves is surrounded by a bright ring-shaped flickering light, which serves as the glare source. Part of the light from the ring is scattered in the eye, including the retina, resulting in the perception that the test field is flickering. A variable amount of counter phase compensation light is then presented in one of the semicircles and the patient must choose the side that flickers more intensely. By adjusting the amount of compensation light, the flicker perception can be eliminated and the stray light modulation from scattered light is compensated for. To obtain the stray light value, this process is repeated several times with different levels of compensation light (Michael et al., 2010, Van den Berg et al., 2013).

## 4 Theoretical basis of corneal transparency

The transparency of the corneal stroma has been studied since the nineteenth century and the general consensus was that the cornea needed to be optically homogenous to be transparent (Schweigger-Seidel, 1869, Von Fleischl, 1880, Ranvier, 1898). The suggestion that collagen fibrils needed to have the same refractive index as their surroundings was stated specifically by Renault (1889). These ideas persisted through the first half of the twentieth century, but measurements of the refractive index of the corneal collagen eventually indicated a significant difference between the collagen and the interstitial matrix (Aurell and Holmgren, 1953). This lead researchers to look for other mechanisms and specifically to the ideas of interference between scattered light waves from ordered crystalline arrays of collagen fibrils (Maurice, 1957). In this section we will describe qualitatively our current understanding of corneal transparency. We will briefly explain what electromagnetic radiation is, how it interferes, and how interference of secondary radiation from the collagen fibrils in the cornea can eliminate light scattering in all directions except forwards.

### 4.1 Electromagnetic radiation

Oscillating electric charges induce a perturbation of the electric and magnetic fields that arise from them that propagates through space. If an electric charge, for example, is moving up and down along an axis with sinusoidal motion, it gives rise to oscillating electric and magnetic fields that, to a first approximation and away from their source, are sine waves. These waves carry energy and can interact with other electric charges, forcing them to move sinusoidally and produce secondary sinusoidal electromagnetic waves.

**Fig. 4:**
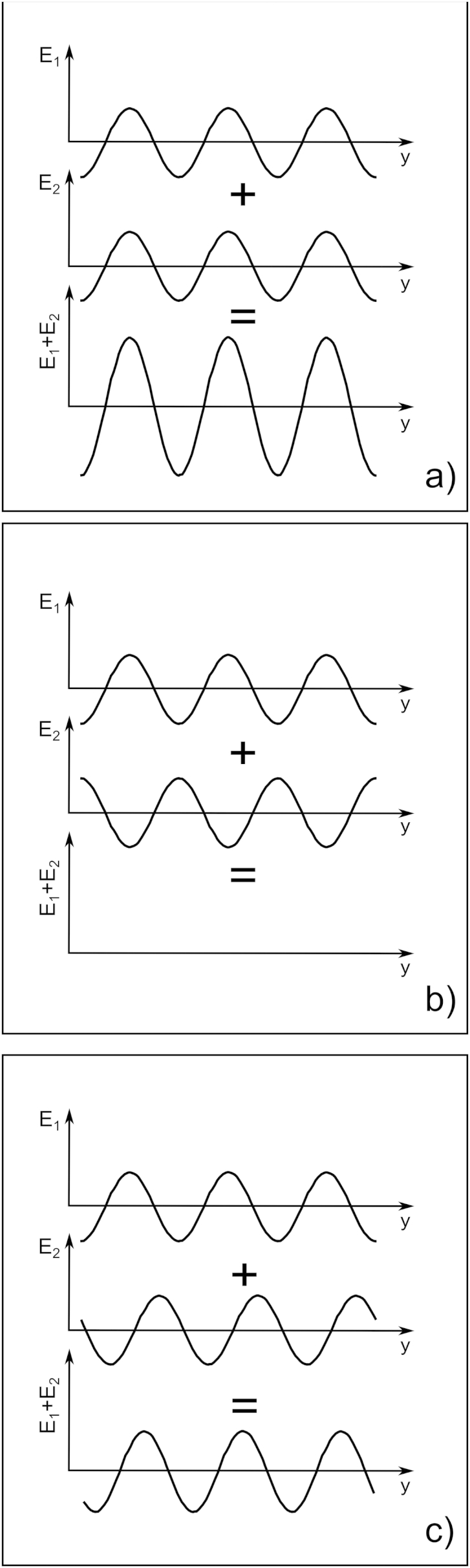
Wave interference of two one-dimensional sinusoidal waves. The two interfering waves are at the top of each panel and the resulting wave, which is a point by point sum of the interfering waves, at the bottom. In (a) the interfering waves are in phase. In (b) out of phase and in (c) they are shifted with respect to each other.

If two electromagnetic waves travel through the same portion of space at the same time they superpose and give rise to interference. Mathematically this corresponds to adding the two waves together, point by point, for every instant. Fig. 4 is an example of possible effects of the interference between the electric field of two one-dimensional electromagnetic waves with the same wavelength and amplitude. If the electric fields from the two waves, E1 and E2, are in phase (with peaks over peaks – Fig. 4a), they give rise to a wave with the same wavelength, but twice the amplitude, when they interfere. If instead they are out of phase (with peaks over troughs – Fig. 4b), when they interfere they cancel out. In general the resulting wave will have, at any one time, the same wavelength as the original waves E1 and E2, but its amplitude will be between zero and twice the amplitude of each original wave, depending on the relative shift between them (Fig. 4c). Although the example described above considers two one-dimensional plane waves, interference happens for all types of waves, also in two and three dimensions.

### 4.2 Interaction of electromagnetic radiation with collagen fibrils

The electric fields associated with electromagnetic waves can excite electrons in matter. Each excited electron oscillates and gives rise to secondary waves which propagate outwards, through space, in all directions, as spherical waves. The wavefronts of these waves can be visualised as concentric spheres, a wavelength apart, whose radii increase in time at the speed of light. As they move through space, these electromagnetic waves interact with all the electrons in the collagen fibril which radiate secondary spherical waves. The superposition of all secondary waves from the excited electrons in the fibril produces a resulting wave that is no longer spherical, but cylindrical. The wavefronts of the cylindrical waves can be visualised as uniaxial cylinders with radii one wavelength apart and sharing their axis with the collagen fibril. The resulting cylindrical waves expand radially away from the collagen fibril at the speed of light. Their wavelength λ is the same as that of the incoming plane wave. The intensity of these waves is inversely proportional to the distance from the fibril, and depends on many factors:

• the radius of the collagen fibril,
• the wavelength of the incoming electromagnetic field,
• the speed at which the waves can move outside and inside the collagen fibril (which is a property of the material and is quantified by its refractive index),
• the direction of oscillation of the incoming electric (or magnetic) field with respect to the axis of the collagen fibril (whether it is perpendicular to it, parallel to it or in any direction in-between – a property described as the polarisation of the incoming electromagnetic field), and
• the direction of propagation of the secondary waves.

**Fig. 5:**
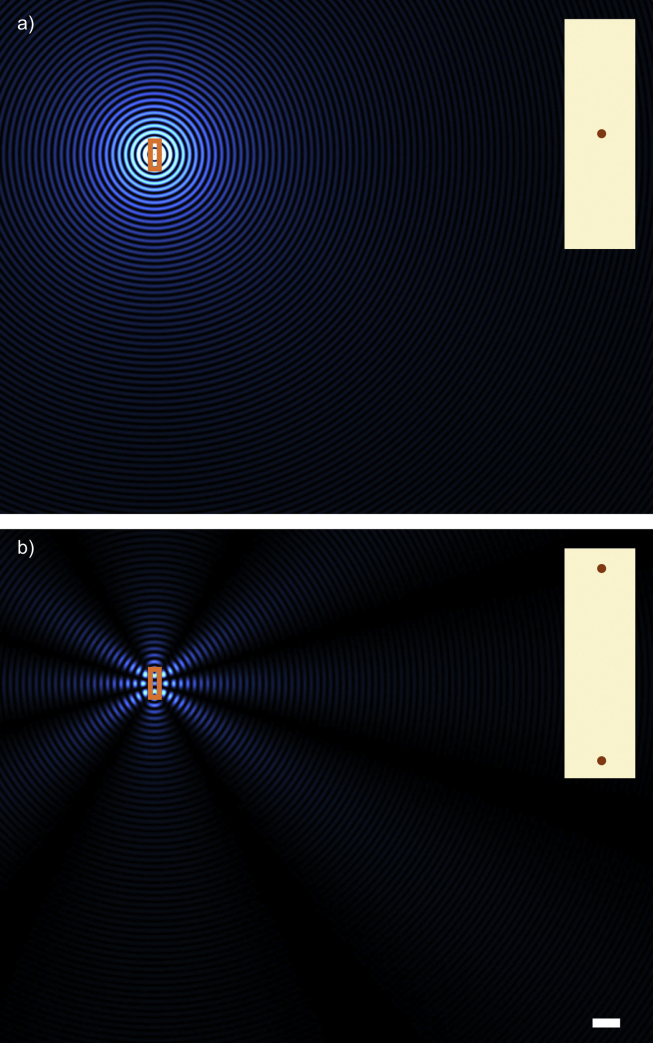
Secondary waves from a single (a) and pair (b) of collagen fibrils. The right hand side insets are a magnified view of the fibril arrangement contained in the rectangle in the main panels. The bar, 1 μm, is in common to (a) and (b).

**Fig. 6:**
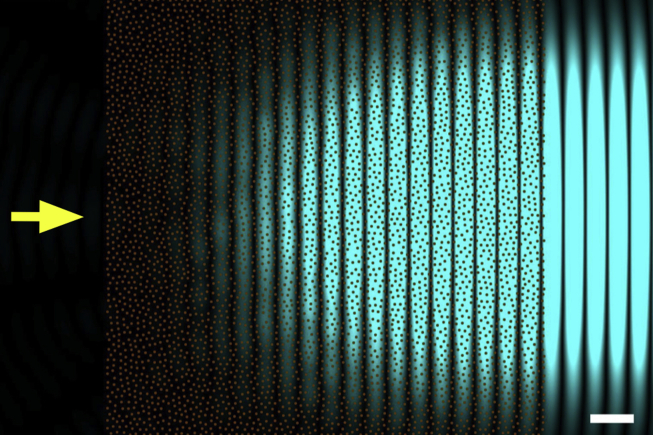
Secondary waves from a collagen fibril distribution presenting short-range order. Primary incoming light is travelling from left to right (yellow arrow), with a wavelength of 500 nm. Collagen fibrils in transverse sections are represented by brown circles. All fibrils have the same diameter of about 31 nm, and no collagen fibrils can be closer than 62 nm. Only the intensity of the secondary radiation arising from the fibrils is shown in blue. No backwards secondary radiation can be seen in the figure. Bar 500 nm.

**Fig. 7:**
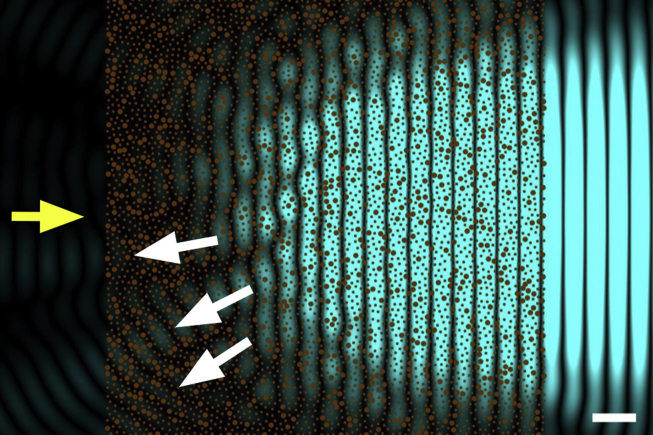
The effect of increased fibril diameters on light transmission. Secondary waves from the same collagen fibril distribution shown in Fig. 6. As before, primary incoming light is travelling from left to right (yellow arrow), with a wavelength of 500 nm. Collagen fibrils in transverse sections are represented by brown circles. 20% of the fibrils were selected at random and their diameter was doubled to 62 nm. The intensity of the secondary radiation arising from the fibrils is shown in blue. Backwards secondary radiation is evident in the figure (white arrows). Bar 500 nm.

**Fig. 8:**
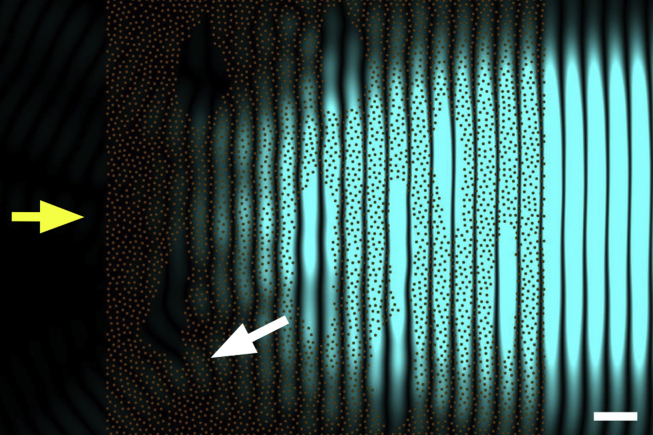
The effect of fibril voids on light transmission. Secondary waves from the same collagen fibril distribution shown in Fig. 6. As before, primary incoming light is travelling from left to right (yellow arrow), with a wavelength of 500 nm. Collagen fibrils in transverse sections are represented by brown circles. Regions devoid of fibrils are now present (lakes). The intensity of the secondary radiation arising from the fibrils is shown in blue. Even in this case, backwards secondary radiation is evident in the figure (white arrow). Bar 500 nm.

An important aspect of the interaction of the incoming electromagnetic waves with a collagen fibril, is that part of the energy of the incoming waves is transferred to the secondary waves and radiated (scattered) in all directions. Since many collagen fibrils are encountered by the incoming waves and each fibril acts as a scatterer, it can be expected that after passing through the whole cornea, very little of the incoming light (Maurice in 1957 calculated about 6%) is left to reach the retina. In other words, we would expect the cornea to be almost completely opaque. The reason why the cornea is not opaque is that, as suggested by Maurice in 1957, the secondary waves from all collagen fibrils interfere with each other in a way that they cancel out in all directions except the forward direction. In practice, although the incoming waves are scattered away almost completely, they transfer their energy to the secondary waves that pass through the cornea. For interested readers, a mathematical description of these interactions can be found, for example, in the papers by Maurice, 1957, Benedek, 1971 and Freund et al. (1986). Here, we can illustrate these ideas non-mathematically by computing this interaction (Fig. 5, Fig. 6, Fig. 7, Fig. 8).

Fig. 5a shows a two-dimensional snapshot of the intensity of secondary radiation scattered from a single collagen fibril (shown in the inset). These are concentric cylindrical waves radiating from the fibril in all directions. As for all the other cases illustrated in Fig. 5, Fig. 6, Fig. 7, Fig. 8, this particular panel was calculated for secondary waves caused by incoming radiation travelling from left to right with polarisation parallel to the fibril axis.^1^ If we take into account the scattered waves from two collagen fibrils (Fig. 5b), we see that they interfere. This simple case shows that, if the wavelength is smaller than the fibril separation as depicted, there can be directions in which secondary radiation cancels out completely and others where they reinforce so that the resulting secondary waves exist only along distinct directions which are related to the fibril separation. This is the case, for example, when X-rays (electromagnetic waves characterised by short wavelengths and invisible to the naked eye) pass through the cornea, and by measuring the angles of these so-called diffracted beams, the fibril separation can be calculated (Meek and Quantock, 2001).

### 4.3 Fibril organisation and transparency

Maurice (1957) proposed an arrangement for the collagen fibrils in the cornea for which secondary radiation would cancel out everywhere except in the forward direction, so that all the energy of the incoming radiation propagates forward through the cornea. The arrangement proposed by Maurice was very simple and consisted of collagen fibrils of uniform diameter on a perfect hexagonal lattice, with the distance between adjacent fibrils being a fraction of the wavelength of the incoming wave. As it turns out, the requirement of a perfect hexagonal lattice for the arrangement of the collagen fibrils is not a stringent one, in fact, to have a transparent cornea, it is sufficient for the distance between adjacent fibril to be constrained. For example, Fig. 6 shows the calculated secondary radiation (painted in blue) emerging from an arrangement of fibrils (represented with brown circles) whose positions are chosen at random but are subject to the restriction that any two fibrils cannot be closer than 62 nm, the centre-to-centre separation at which two human collagen fibrils would touch. This type of fibrillar arrangement is said to possess short range order and this property is essential to corneal transparency. In fact, as seen in the figure, once the secondary radiation from all fibrils is added together, backwards radiation cancels out completely and only forward radiation remains, which carries the energy lost by the incoming radiation on through the cornea.

Because the intensity of the scattered wave from a fibril depends on the amount of collagen within the fibril (and therefore its radius), perfect backward destructive interference cannot be achieved from collagen fibrils whose diameters are not constant, as in Fig. 7. The fibrillar arrangement in Fig. 7 is identical to that of Fig. 6, but with 20% of the fibrils having a diameter twice the normal size. This is sufficient for backward radiation to arise (white arrows). To understand how this can happen, consider Fig. 4b and imagine the two out-of-phase waves in the panel having different amplitudes: once they are summed together they will not be able to cancel out any longer. The same would happen for the cylindrical secondary waves of different amplitudes coming from the collagen fibrils: they would not be able to interfere destructively in the backward direction. Corneal opacity due to this mechanism arises when adjacent fibrils join randomly together to form larger fibrils with varying diameters. Corneal structural defects of this type are associated, for example, with genetic mutations involving proteoglycans (Chakravarti et al., 1998) which, ultimately, seem to have an effect on the repulsive forces between adjacent fibrils and allow them to fuse together.

If the cornea swells much above its physiological hydration it begins to scatter significant quantities of light and to lose its transparency. The gel pressure that causes this swelling arises from net electrostatic charges within the stroma. Most of the electrostatic charge is contributed by the acidic groups of the glycosaminoglycans. The other contribution to net stromal charge arises from the temporary complexing of free chloride ions to ligands within the stroma (Hodson, 1997). An endothelium-based pumping mechanism maintains corneal hydration which would otherwise increase due to the stromal gel pressure (Stiemke et al., 1995). If this mechanism fails and the cornea swells, regions devoid of collagen fibrils (termed lakes) impair corneal transparency (Clark, 2004, Meek et al., 2003). In Fig. 8 the fibril arrangement is identical to that of Fig. 6, but here there are regions devoid of fibrils. If fibrils were present in these regions, they would contribute to cancelling out the overall secondary radiation in the backward direction as in Fig. 6, but since they are missing, they cannot do so and backward radiation cannot be cancelled out completely. However, for backward scattering to arise, these regions have to be separated by distances comparable with the wavelength of the incoming light. The backscattered light shown in Fig. 7, Fig. 8 is only a small fraction of the secondary radiation emerging from the fibrils. However, in real corneas, the incoming light is scattered by many more fibrils than those shown, so that the overall loss of intensity becomes significant and corneas with such structural defects exhibit impaired transparency.

The explanation for cornea transparency presented above is based on the approximation that the secondary electromagnetic radiation scattered by any collagen fibril will not interact with other fibrils of the cornea, but will travel undisturbed to the retina. This cannot be the case in practice, and attempts have been carried out to include multiple scattering by applying photonic band structure theory to the cornea which provides a way to account for multiple scattering (Ameen et al., 1998). However, at present, it has been successfully applied only in the approximation that the collagen fibrils in the corneal stroma are arranged as a perfect hexagonal lattice, which we know not to be the case. The use of other computational methods, such as the finite difference time domains method (FDTD – Yee, 1966) may prove a more successful way to tackle the problem of multiple scattering by the collagen fibrils in the cornea, even in the case of randomly arranged fibrils with varying diameters.

We should point out that the energy scattered by each fibril when unpolarised light falls on it is inversely proportional to the third (Maurice, 1962) or fourth (Van den Berg and Tan, 1994) power of the wavelength of the incoming light. Theoretical considerations suggest this to be true for whole corneas in which the fibril arrangement has short range order (as in Fig. 7). Since experimental measurements from rabbit corneas in the visible spectrum have found a λ^−3^ dependence for the transmission of the scattered light (Farrell et al., 1973), we can take it as an indication that the fibril arrangement seen in micrographs, which has short range order (Fig. 12b, inset) is a very good representation of the fibril arrangement present in the corneal lamellae in vivo (Farrell et al., 1973). We should also point out that any alternative model for the packing of fibrils in the cornea must obey this wavelength dependence.

**Fig. 9:**
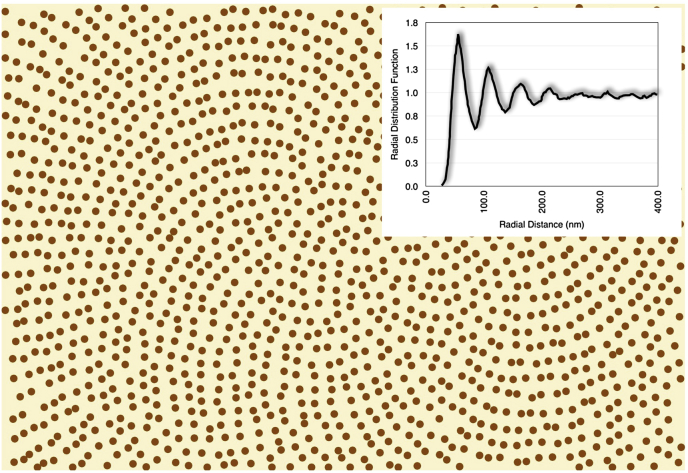
Disordered Fibonacci quasi-crystal arrangement of fibrils. This type of quasi-crystal is deterministic over a long range. The radial distribution function (inset) plots the number of fibrils per unit area (normalized by dividing by the fibril number density in the image) separated by the distance shown on the horizontal axis. This is very similar to that seen from electron micrographs from the cornea (cf. Fig. 12b inset). Modified from Doutch (2009) with permission of the copyright holder.

J.J. Doutch (2009) carried out his doctoral thesis within our research group and, following a suggestion by S. Chakravarti, modelled the arrangement of the collagen fibrils in the cornea by treating them as Fibonacci quasi-crystals, in which the fibrillar arrangement is similar to that of the seeds in sunflower heads (Vogel, 1979). Such a photonic crystal-like fibril arrangement (Fig. 9) has the advantage of being easily treatable from a theoretical point of view and Doutch found that not only does its appearance closely resemble the collagen fibril organization seen in micrographs, but also that the wavelength dependence of the predicted transparency matches very closely the experimental one. However, it is very difficult to see how a Fibonacci quasi-crystalline fibril arrangement could arise spontaneously in a cornea, especially in the light of non-systematic interactions between collagen fibrils and proteoglycans (see below – Lewis et al., 2010). Since a corneal model obtained by randomly distributed collagen fibrils has equally good predictive power (see below and Fig. 12, Fig. 13), such a model, being simpler, should be preferable.

### 4.4 Contribution of stromal cells

Keratocytes are the main cells within the corneal stroma and they serve to maintain the slow turnover of the connective tissue matrix, synthesizing collagen molecules and proteoglycans and producing matrix metalloproteinases. Other cells are also present, including transient bone marrow-derived cells, macrophages and dendritic cells (Hassell and Birk, 2010). The keratocytes are regarded as being quiescent but can be activated after injury generating myofibroblast precursors that can sometimes persist and cause corneal scarring due to their own opacity as well as their production of fibrotic tissue (Torreicelli and Wilson, 2014). Keratocytes themselves are transparent except for their nuclei.

To date, the attempts to explain the observed transparency of the cornea have focussed only on the arrangement of the fibrils of the stroma as described above, and have ignored the presence of the stromal cells. David Maurice and others gave the justification that the keratocytes are sparsely distributed and are thin in the direction of the passing light. However, we now know that these cells could contribute up to 15% of the total stromal volume (Huang and Meek, 1999) so an alternative explanation for cellular transparency was put forward by Jester and his colleagues (Jester et al., 1999, Jester, 2008). They postulated that stromal cells contain cytoplasmic molecules called corneal crystallins that act in much the same was as crystallins in the lens. These crystallins are the principal UV-absorbing proteins alluded to earlier, but they have a dual role in that they change the refractive index of the cell cytoplasm to better match the extracellular matrix and thus eliminate scattering (Chen et al., 2013). They are thought to limit backwards scattering by forming a structural unit that promotes short-range order (Piatigorsky, 1998). In the mammalian cornea, the most likely candidates are aldehyde dehydrogenases, particularly ALDH3A1, that was first detected in the bovine cornea by Holt and Kinoshita (1973) and later confirmed by others (Alexander et al., 1981, Silverman et al., 1981, Chen et al., 2013).

Confocal microscope studies of corneal wound healing suggest that the activation of the keratocyte cells could be responsible for the increase in scattering (Jester et al., 1999, Moller-Pedersen, 2004). Any change in cytoplasmic content when the keratocytes transform to myofibroblasts is likely to alter the refractive index, and this would then contribute to the increase in light scatter. Recent measurements from our group have shown that the refractive index of the keratocyte cytoplasm matches that of the stroma as a whole, whereas, upon activation, the refractive index falls. Modelling of Mie scatter shows that this change in refractive index is sufficient to significantly increase light scattering by these cells (SJ Gardner, N White, J Albon, C Knupp, CS Kamma-Lorger and KM Meek, unpublished results).

## 5 Molecular mechanisms of corneal transparency

Two of the most important structural factors for corneal transparency are the uniformity of the diameters of the collagen fibrils, and the restriction in the range of distances between adjacent collagen fibrils. Below, we will discuss how collagen molecules and proteoglycans interact in the cornea to achieve these characteristics.

A clue to how the precise diameters of the collagen fibrils are determined in the cornea came from an analysis of the collagen content of these fibrils in chicken embryos (Birk et al., 1986). It was found that corneal fibrils were a blend of type I and type V collagens, but unlike in other tissues, the collagen V to collagen I ratio was noticeably higher in corneal stroma (Birk et al., 1990). Both collagen types, when purified, could assemble in vitro to form fibrils, but in this case type I collagen formed fibrils of varying diameters, while type V formed small fibrils. It was also found that in corneal stroma, type V collagen molecules retained their amino-terminal non-collagenous domain. In vitro experiments aimed at creating fibrils using purified collagen I and collagen V molecules, showed that the type V collagen molecule terminal domain was necessary to regulate the diameters of the formed fibrils (Birk et al., 1990). In view of this, it seems likely that without the terminal domains, the amino acid distribution along the type V and type I collagen molecules is such that the collagen molecules experience electrostatic and hydrophobic forces that drive them to assemble side by side, with a 67 nm axial shift, and form large heterotypic fibrils. However, the bulky non-collagenous domains of type V, when present, introduce small mismatches in the alignment of the type I and type V collagen molecules. These mismatches accumulate as more type V molecules are incorporated, to a point when no new molecule is able to join the fibril, because it cannot create electrostatic and hydrophobic bonds strong enough to maintain the molecule in place. In this model the amount of collagen V would regulate the number of molecules able to form a fibril and therefore its size: the less collagen V is present, the larger the fibril diameter. This has been seen to be the case when studying genetically defective mouse in which collagen V content was reduced (Hassell and Birk, 2010). However, it was noticed that, in vitro, the fibrils formed by collagen I and collagen V are larger than those found in vivo. This indicates that there may be additional mechanisms such as microfibrillar coiling (Raspanti, 2010) or collagen-bound disaccharides (Harding et al., 1980) that regulate fibril diameter.

Early in vitro studies of reconstituted collagen fibrils, showed that the presence of lumican and decorin protein cores had an effect on the size of the collagen fibrils: fibrils reconstituted in the presence of the protein cores of the proteoglycans were significantly thinner than the fibrils reconstituted in the absence of the cores (Rada et al., 1993). These findings were confirmed in studies of mutant mice unable to produce the necessary amount of lumican, decorin and biglycan in the cornea (Chakravarti et al., 1998, Chakravarti et al., 2006, Zhang et al., 2008). Three-dimensional electron tomographic studies (Parfitt, 2011), showed that, at least in the case of lumican-deficient mice, the increase in fibrils size can be attributed to a lateral association of normal sized collagen fibrils. In general, the electrostatic and hydrophobic forces that drive the collagen molecules together to form fibrils may be strong enough to guide and maintain the lateral association of two or more fully formed collagen fibrils. In practice, this would not happen when the protein cores of the proteoglycans are present on the surface of the collagen fibrils, because their bulk would prevent adjacent fibrils from coming into contact and establish the necessary bonds.

**Fig. 10:**
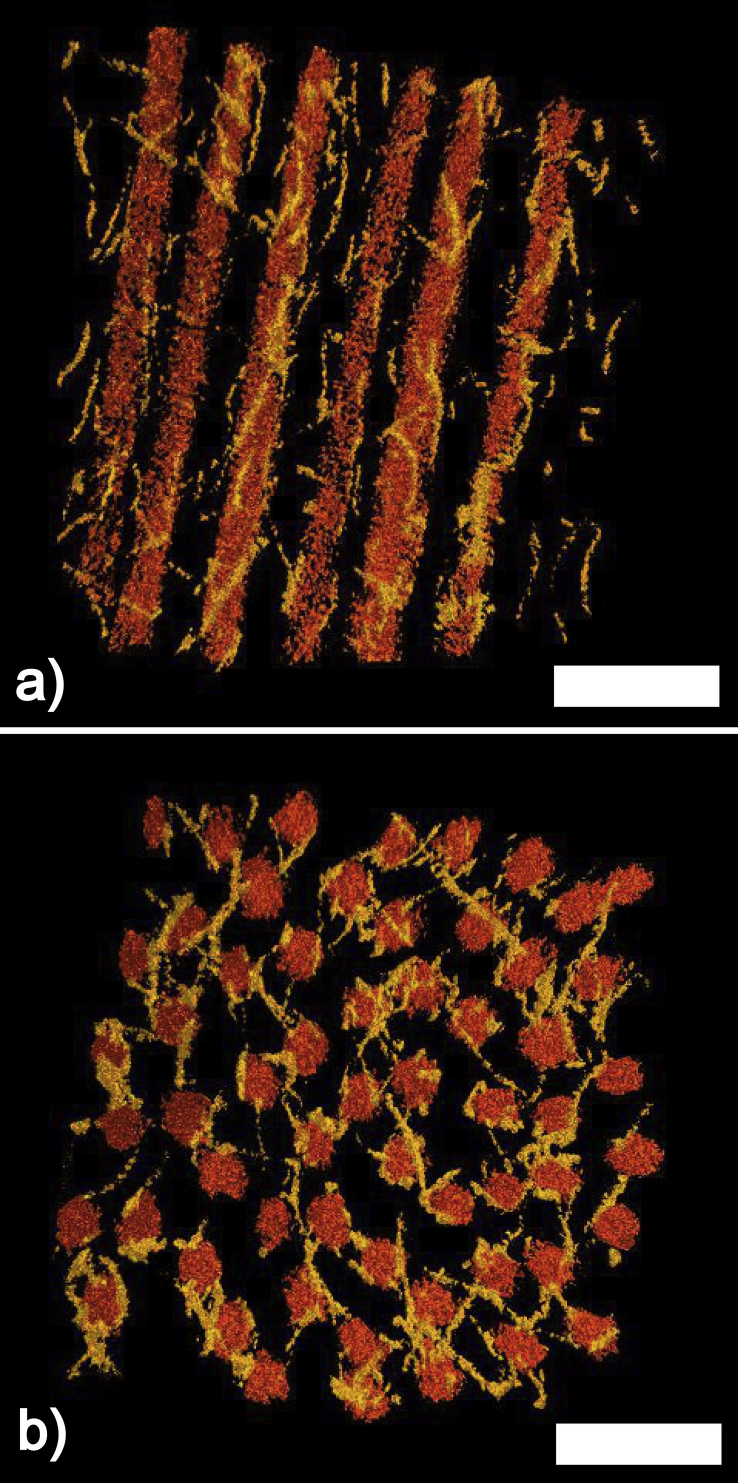
Three-dimensional corneal stroma reconstruction.(a) Longitudinal section. (b) Transverse section. In both panels the collagen fibrils are depicted in orange and proteoglycans in yellow. Bars 100 nm. Modified from Lewis et al. (2010) with permission of the copyright holder.

An attempt at understanding the molecular mechanisms that were responsible for keeping the collagen fibrils at defined distances was carried out by Maurice in 1962 and Hart and Farrell in 1969. The models they proposed were based essentially on the collagen fibrils in the corneal stroma being connected to six adjacent fibrils by proteoglycans at regular axial intervals. The defined lengths of the proteoglycans would force the fibrils on a perfect hexagonal lattice, which would guarantee corneal transparency. More recently, Müller and collaborators (2004) modified the model by Maurice, on the basis of electron microscopical observations and theoretical considerations on the published dimensions of the proteoglycans. In the model by Muller et al., the collagen fibrils still lie on a perfect hexagonal lattice, but they are kept in position by proteoglycans linking next nearest fibrils as opposed to adjacent fibrils as in in the model by Maurice. In 2010, we proposed a new mechanism on how the inter-fibrillar distances are maintained by the proteoglycans (Lewis et al., 2010). Our model was based on three-dimensional electron tomographic reconstructions of bovine cornea (Fig. 10a and b). From the reconstructions, it was evident that the collagen fibrils were not laying on a perfect hexagonal lattice, and also that there was nothing systematic in the geometry of the connections of adjacent or next nearest collagen fibrils. What was evident, however, was that the distribution of distances between adjacent fibrils was narrow. It was therefore proposed that the distances between adjacent collagen fibrils are a consequence of the balancing of two opposing forces acting on the fibrils brought about by the proteoglycans. In this model, both opposing forces depend on the separation between the fibrils, and the equilibrium distance between two fibrils is reached when the attractive forces between them cancel out the repulsive forces. The repulsive forces between the fibrils are due to the pressure exerted by water molecules attracted between the fibrils via the Donnan effect.^2^ The attractive forces are due to those proteoglycans that connect two or more fibrils. These proteoglycans would be subject to thermally driven conformational changes so that their glycosaminoglycan chains will experience a force that prevents them from assuming a fully extended conformation and which brings their terminal ends, and the collagen fibrils attached to them, closer together. These forces are equivalent to the molecular forces arising within a stretched rubber band, which are responsible for the rubber band's elasticity (Feynman et al., 1963). Another characteristic of this model is that the connections between adjacent fibrils by the proteoglycans are not necessarily permanent, but can be broken and reformed continuously, as long as a sufficient proportion of connections are present at any one time to guarantee attractive forces between the fibrils. This would endow the whole cornea with a less rigid and more fluid overall structure which would make the cornea more resilient to deformations due to external stresses and would facilitate the transport of molecules and nutrients across the cornea.

**Fig. 11:**
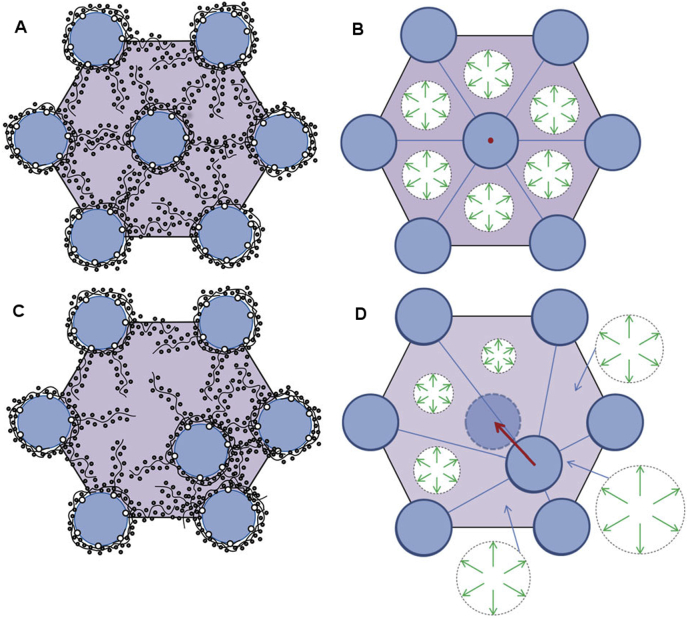
The electrostatic restoring force mechanism which acts on fibrils to maintain the lattice organization. The mechanism is illustrated for the case where all fibrils in the lattice are held fixed in their lattice positions and a single fibril is displaced relative to its lattice position. (a) Shows a set of fibrils in a regular lattice arrangement with their associated GAG chains and a distribution of mobile ions. In this situation, which corresponds to normal physiological conditions, the osmotic pressure is essentially uniform. (b) Depicts the osmotic pressure in each subcell around the undisturbed fibril. The osmotic pressure in each subcell exerts a force on the fibril but because the osmotic pressure is uniform, all six forces balance and the net force is zero, as indicated by the red dot. (c) Shows a fibril displaced from its lattice position. The GAG chains attached to the fibril move with the fibril and the GAG fixed charge density and mobile ion concentration increase in advance of the fibril displacement and reduce behind it. (d) Indicates the resulting osmotic pressure in each of the subcells, which is higher where GAG fixed charge density has increased and lower where it has reduced. The forces exerted by each of the subcells now has changed magnitude with the result that a net force acts on the fibril with a direction oriented towards the original lattice position. This restoring force is shown as a red arrow. Figure and caption reproduced from Cheng and Pinsky (2013) with permission of the copyright holder.

More recently, Cheng and Pinsky (2013) performed a theoretical analysis of the forces exerted by the proteoglycans to maintain the short range order of the collagen fibrils in the cornea. They sub-divided the PGs into two populations, one coating the collagen fibrils and a second forming bridges between adjacent fibrils. They concluded that electrostatic forces due to the PGs coating the fibrils play little part in fibril spacing except when fibrils get very close, at which point these PGs serve to prevent fibril fusion. On the other hand, the ions attracted by the unbalanced charge on the glycosaminoglycan chains of the interfibrillar PGs play a dominant role in keeping the fibrils on optimal lattice positions, by acting as restoring forces, and the authors showed that these, by themselves, can reproduce the image distribution of fibrils seen in the electron microscope. Their mechanism, illustrated in Fig. 11, operates regardless of the direction of the perturbation and regardless of whether GAG chains are part of a duplex or are independent. When any fibril undergoes a dynamic excursion from its lattice position, the electrostatic restoring forces act to restore the fibril. This mechanism would also be relevant to corneal development and to the establishment of the stromal matrix. In this respect it is worth pointing out that in a study of over 40 animal species, including fish, birds, amphibians and mammals, Meek and Leonard (1993) reported that, even though there was a wide inter-species variation of fibril diameters and centre-to-centre fibril spacings, the volume fraction (i.e. the proportion of the tissue occupied by the hydrated fibrils and hence by the interfibrillar GAGs) was constant. This implies that the larger the fibril diameter in a species, the larger the space occupied by the proteoglycans. Because both polyanionic charge per unit volume and tissue hydration are constant between species, regardless of the PG composition (Scott and Bosworth, 1990, Gyi, 1988), they proposed a model in which the fixed charges on GAGs are synthesised in proportion to the total fibrillar collagen (types I and V) (Meek and Leonard, 1993); the larger the collagen fibrils, the greater amount of fixed charge in the interfibrillar space, therefore the further apart the fibrils. This idea is in agreement with the model of Cheng and Pinsky (Fig. 11); if the collagen fibrils were larger in diameter and the interfibrillar space was constant, the repulsive forces generated by the GAGs and their associated ions would be increased, which would increase the swelling pressure and push the fibrils further apart in order to achieve equilibrium.

**Fig. 12:**
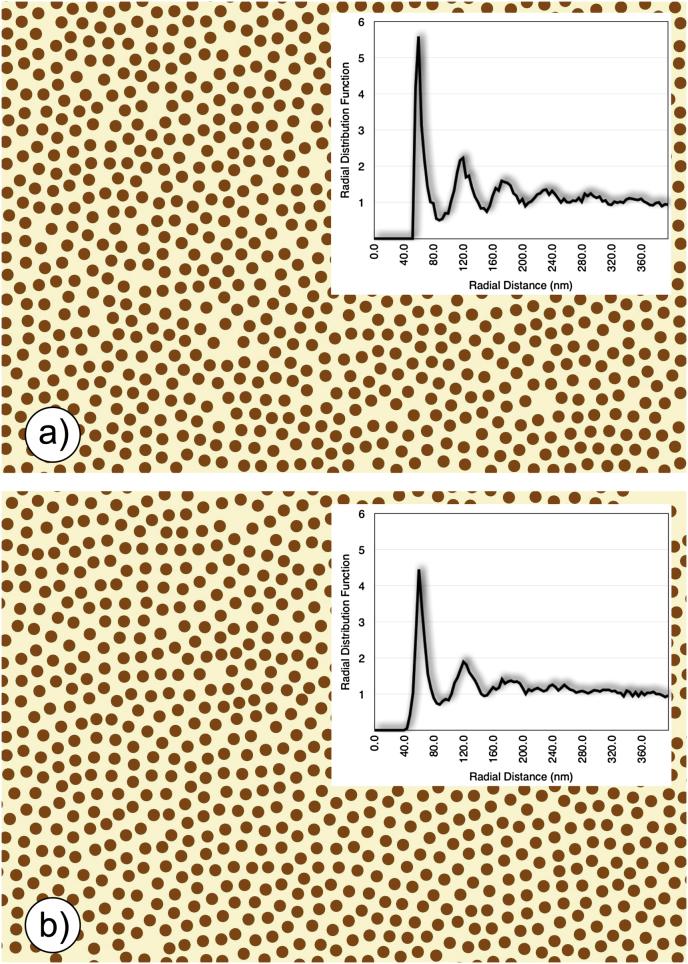
Collagen fibril positions and corresponding radial distribution function (inset) from (a) a computer simulation and (b) a transmission electron micrograph of a rabbit cornea. The radial distribution function plots the number of fibrils per unit area (normalized by dividing by the fibril number density in the image) separated by the distance shown on the horizontal axis.

**Fig. 13:**
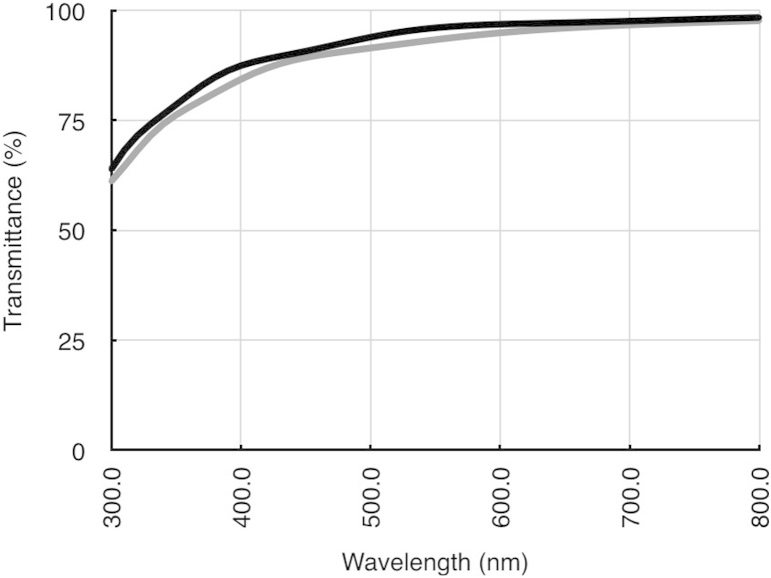
Calculated light transmission in the rabbit cornea as a function of wavelength for the collagen fibril distributions represented in Fig. 12a (grey curve) and Fig. 12b (black curve). Both curves were calculated using the direct summation of fields method (Freund et al., 1986), using other parameters taken from rabbit cornea (corneal thickness 360 microns, refractive index of collagen 1.355, refractive index of ground substance 1.420, collagen radius 19.4 nm, fibril density 220 fibrils per square micron).

Recent preliminary unpublished work by Knupp aimed at elucidating the role of keratocytes in corneal fibril deposition suggested that there may be another type of “attractive” force acting on the fibrils that would prevent them from drifting apart. During his study, Knupp was trying to ascertain if the distribution of the positions of the collagen fibrils in the cornea could be explained by a random deposition of the collagen fibrils in the underlying matrix by the keratocytes. A computer simulation was set up in which collagen fibrils were added at random positions in a confined region, with the condition that a new fibril would be added only if its distance from any other fibril already present was more than 56 nm (the average separation between adjacent fibrils in rabbit corneas). The exclusion zone around the fibrils was mimicking the repulsive action of the proteoglycans which would prevent the fibrils from coming too close to each other. Bearing in mind how simple the simulation was, the appearance of the corneal fibril distribution obtained in this way (Fig. 12a) was remarkably similar to that taken from an electron micrograph of a rabbit cornea (Fig. 12b), as indicated by the calculated radial distribution functions of the fibril positions (insets) and the predicted transmission as a function of wavelength (Fig. 13). This supported the hypothesis that the appearance of a cornea could be explained by a random deposition of fibrils with an exclusion zone around each one, but there were other implications arising from this simple theoretical experiment. In fact, no specific attractive forces between the collagen fibrils were introduced in the simulation, which implied that the attractive forces had to come from elsewhere. Since the only constraint in the simulation, apart from that of the minimum distance between adjacent fibrils, came from the fact that the fibrils were restricted to be in a closed region, it must be that the “pressure” exerted by the walls of the region was enough to keep the fibrils close to each other. This suggested that there may be another source of attractive forces between fibrils in the cornea which come from the pressure exerted by the adjacent lamellae. In this case, the fibrils at the interface between lamellae would be pushed inwards, creating a cascade effect that ultimately would hold all fibrils within any lamella close to each other. However, how important the overall contribution of this mechanism is in maintaining the arrangement of the collagen fibrils in the cornea remains to be determined.

## 6 Causes of light scatter in corneal pathologies

A number of corneal pathologies increase light scatter, often to the extent that a lamellar or penetrating keratoplasty is required. In many cases the cornea appears cloudy or opaque, but the underlying cause of this light scatter can have several different structural bases. Opacities associated with corneal dystrophies (granular dystrophy, lattice dystrophy, macular dystrophy, Schnyder dystrophy) are caused mostly by different kinds of stromal deposits. Such deposits cause local changes in the refractive index of the stroma, and these refractive index discontinuities, if comparable with the wavelength of light, lead to increased light scatter. Matrix changes also occur but these contribute less to the observed light scatter. A similar effect occurs in corneal oedema (bullous keratopathy, Fuch's dystrophy) but in this case there are local decreases in refractive index caused by pooling of fluid into so-called “lakes” (Casadessus et al., 2012) (Fig. 8). The influx of fluid into the stroma also leads to disruption of the fibril organisation and thus to the reduction in destructive interference of scattered light. Corneal opacity during corneal wound healing, or transitory haze following refractive surgery or corneal cross-linking, is caused by activated corneal keratocytes (Connon et al., 2003, Moller-Pedersen, 2004). Once activated, these cells have a lower refractive index than the surrounding connective tissue. On the other hand, the opacity of corneal scars is a result of improper remodelling of the connective tissue matrix; collagen fibrils are wider than normal and poorly ordered in scar tissue (Boote et al., 2013). The presence of similar oversized collagen fibrils, probably formed by fibril fusion (Fig. 7), is also a major cause of corneal light scatter seen in many of the mucopolysaccharidoses, though there is often accompanying deposition of stored proteoglycans with cells or within the matrix that also contributes. Thus haze or opacity within the cornea can have multiple causes, and it is often incorrect to attribute the light scattering to a single structural change that may be observed within the tissue.

## 7 Future directions

The most striking advance in corneal structural studies over recent years has been the rapid development of new imaging technologies, particularly those that allow structures to be examined in three dimensions. The ideal scenario would be to visualise corneal structure in three-dimensions in vivo. To some extent, confocal microscopy allows this, but the resolution is poor. High resolution polarization-sensitive optical coherence tomography (PS-OCT) may be used in vivo, and the approach has also allowed depth-resolved visualisation of the cornea from which information about collagen fibrils can be derived (Pircher et al., 2011). The authors point out that PS-OCT might be useful to detect early changes of fibril orientation, prior to any macroscopically visible structural changes, and therefore might be a valuable tool for early diagnosis of keratoconus. Higher resolutions are currently only available *ex vivo*. While X-ray and visible light scattering are still the best methods to determine structure at the molecular level, non-linear SHG and related microscopies have recently provided invaluable new data on the 3-D lamellar organisation, much more than has been possible using conventional and polarised light microscopy. Soft X-ray microscopy is also being developed at various synchrotron light sources worldwide. Originally it was believed that this technology would be impossible due to the inherent difficulty of focussing X-rays, not to mention specimen damage, but the techniques have now been developed to a resolution somewhere between the cellular and the molecular level, whilst pulsing the radiation can reduce damage to the sample. The penetration depth of soft X-rays is ideally suited to imaging intact cells, though at present it is limited to cells with a thickness of only a few microns. The primary advantage of a soft X-ray microscope is its ability to form the highest spatial resolution images of thick hydrated biological samples in a near-native environment, without the time-consuming sample preparation needed for electron microscopy.

An exciting new development has been the introduction of block face scanning electron microscopy. This utilises not the inelastically scattered electrons which show surface topography in conventional scanning electron microscopy (SEM), but the elastic backscattered electrons after the tissue has been embedded and stained with heavy metals in a way similar to conventional transmission electron microscopy (TEM). The two-dimensional image obtained in this way approaches that seen using TEM and allows visualisation of individual corneal collagen fibrils, sub-cellular organelles etc. The principle is that, after the image has been acquired, the surface of the tissue is removed and another image taken, thus producing large data sets from different depths in the tissue. There are currently two systems for block face scanning SEM, Focussed Ion Beam (FIB)-SEM where, after recording an image of the surface of the block, that surface is etched away in the microscope to expose the fresh surface immediately below, or Serial Block Face (SBF)-SEM where an ultramicrotome is built into the microscope and automatically cuts away the surface after imaging, to reveal the next layer down. Both methods allow one to image large volumes of tissue and to carry out three-dimensional reconstructions at the ultrastructural level.

These new techniques will soon be applied to a range of structural studies. SBF- and FIB-SEM have already shown in detail how cells in chick cornea organise the connective tissue matrix that they synthesize (Young et al., 2014) and we have also been using it to move forward our understanding of how the human corneal stroma is formed in utero. It will also have applications in increasing our understanding of cell–matrix interactions and cell–cell communication processes in normal cornea as well as in pathological conditions such as keratoconus.

Another outstanding unknown is exactly how lamellae interweave throughout the cornea. This has important surgical implications, for example how big bubbles form where they do within the cornea, and what causes ectasia either in keratoconus or following some cases of LASIK surgery. A detailed understanding of collagen 3D structure will also help to explain the biomechanical properties of the tissue. This is of some importance to those trying to develop artificial corneal replacements, as it is necessary to try to replicate both the transparency as well as the strength and shape of the original tissue (Ruberti and Zieske, 2008). Currently, trials have been carried out on partial thickness grafts using synthetic collagen gels (Fagerholm et al., 2010), but at present it appears that these gels are acting as temporary scaffolds to allow remodelling of the tissue. For their use as scaffolds for penetrating keratoplasty or for permanent corneal replacements they will need to be refined to replicate many of the biomechanical properties of the cornea. Finally, we still do not fully understand what maintains the aspherical shape of the cornea, what is the structural basis of corneal astigmatism, and exactly how the cornea integrates with the sclera at what appears to be a quite complex limbal region. Many of these questions should be answered in the coming years.

The general basis of corneal transparency outlined above is accepted in principle, although the time is ripe now for modelling corneal transparency as a whole, simultaneously considering not only the collagen fibrils in all the lamellae, but also all the cells that the light encounters along its path. Structural data from real corneas can now be collected using modern techniques such as three-dimensional block face scanning electron microscopy and modern computers are fast enough for transparency predictions to be obtained by solving Maxwell's equations for the collected data. But looking forward, perhaps the most exciting development with regard to corneal transparency is not understanding its causes, but rather developing treatments to restore it. Currently, clinical trials are in progress to use stem cell therapy in corneal wounds. In animals, it has previously been demonstrated how corneal stem cells can restore tissue transparency (Du et al., 2009, Syed-Picard et al., 2015). If the clinical trials are successful and it is found that such treatments can be used in humans, it will be important to understand how they are working at the structural level in order to improve treatment methods and to ensure that the effects can last.

## Footnotes

1 For other polarisations of the incoming wave, the intensity of the scattered waves would depend on their direction of propagation. This, however, would not change the principles illustrated in what follows, and we will concentrate on a parallel polarisation mode for simplicity.

2 The negative charge on the proteoglycan's glycosaminoglycan chains would attract positively charged ions that in turn would attract water molecules by osmosis.
